# Development, stabilization and the shaping of ageing trajectories

**DOI:** 10.1098/rstb.2025.0331

**Published:** 2026-11-12

**Authors:** Miria Ricchetti

**Affiliations:** Institut Pasteur, Université Paris Cité, Molecular Mechanisms of Pathological and Physiological Ageing Unit, UMR3738 CNRS, Paris, France

**Keywords:** ageing, development, cell plasticity, chromatin remodelling, stress, regulatory pathways, exposome

## Abstract

Ageing integrates genetic, epigenetic, developmental and environmental influences across the lifespan, and reflects both damage accumulation and a progressive reduction of homoeostatic and adaptive capacity, narrowing the organism's functional range. These processes reflect the long-term behaviour of systems configured during development. Early-life processes define cellular, structural, endocrine and epigenetic landscapes that shape later-life trajectories, and are modulated by the exposome over time. Beyond the developmental origins of the health and disease framework, development more broadly shapes ageing. Developmental and ageing processes share molecular pathways, and developmental regulatory architectures can be maintained, repurposed or dysregulated later in life. Comparative biology further indicates that lifespan is associated with developmental tempo and long-term maintenance strategies, whereas organisms that retain plasticity and regenerative capacity often display attenuated ageing. Ageing appears linked not simply to development itself, but to the transition from developmental plasticity to stabilization. Ageing becomes more likely when development culminates in highly specialized, structurally stable and weakly renewable systems, which maximize function at the cost of reversibility and repair. This framework is particularly relevant to the brain, where development establishes long-lived, weakly renewable architectures. Ageing thus reflects the interactions between developmental constraints, lifelong environmental exposures and the stabilization of development.

This article is part of the theme issue ‘Developmental circuits in ageing’.

## 1. Introduction

Ageing is unique in biology because it links what an organism is genetically and epigenetically to what it once was as an embryo, and to how the environment (or exposome) has shaped it over time. In that sense, ageing cannot be readily reduced to chronological time. Rather, it reflects a gradual reduction in the capacity of biological systems to maintain homoeostasis and adapt to internal and external stress, resulting in a narrowing of their functional range [1]. Although the drivers of ageing have not yet been fully established [2], ageing is strongly associated with the combined burden of damage and the declining ability to buffer, repair and compensate for it [3]. This adaptive capacity is not uniform across organisms or tissues, but is shaped by developmental processes that define the structural, cellular and regulatory context within which homoeostasis operates throughout life, thereby setting the conditions within which ageing trajectories unfold.

This view of ageing as a dynamic process shaped by the interaction between damage, adaptive capacity, developmental history and environmental exposure has gained strength as the biology of ageing has moved beyond viewing it as a uniform, predetermined process. Ageing can be accelerated, as observed in progeroid syndromes or chronic pathological conditions, but can also be delayed through genetic, nutritional or pharmacological interventions [4–6]. Ageing is thus better understood as a dynamic trajectory rather than a fixed outcome.

A substantial body of work indicates that development is one of its major determinants. The developmental origins of health and disease (DOHaD) framework established that fetal and early postnatal environments can induce durable changes in organ structure, endocrine regulation, metabolism and epigenetic state, which persist into adulthood and influence adult physiology and disease susceptibility [7–10]. Yet this framework captures only part of the relationship between development and ageing. Beyond environmental programming, development establishes long-lived biological constraints. It determines tissue architecture, cellular composition, stem cell pools and their niches, and gene regulatory and epigenetic states that can persist for decades. These constraints define the range of possible system responses over time, thereby shaping both the direction and the flexibility of ageing trajectories. Ageing, therefore, unfolds within a biological system whose properties were largely defined early in life.

This review examines this broader relationship. Ageing has been interpreted through diverse theories, including those emphasizing damage accumulation and evolutionary trade-offs, and models proposing that ageing reflects the persistence or dysregulation of developmental and growth-related processes beyond their optimal temporal window [11–14]. Here, we do not adopt a programmatic view of ageing, nor do we consider ageing as a direct or inevitable outcome of development. Rather, we focus on how developmental processes establish constraints that shape ageing trajectories, how the progressive transition from developmental plasticity to developmental stabilization may predispose to later-life decline, and how these principles apply across systems, with particular emphasis on the brain. In this context, developmental stabilization refers to the progressive restriction of plasticity, regeneration and regulatory flexibility as tissues achieve functional specialization (box 1). This transition marks a shift from a highly plastic, adaptive state to a more stable but increasingly constrained regime. Within this framework, ageing does not result from the continuation of development, but from the operation of shared regulatory systems within this stabilized and progressively constrained biological state, which together define the trajectory of functional decline over time. In other words, the same regulatory pathways that operate during development may continue to function later in life, but within cells and tissues that are more differentiated, less plastic and less regenerative, so that their activities and consequences differ from those during development.

Box 1.
**Key concepts linking development and ageing**

*Exposome.* The totality of environmental exposures experienced throughout life, from the prenatal period onwards, including physical, chemical, nutritional, psychosocial and biological influences. In this essay, the exposome is considered to act on a biological system whose properties have already been shaped by development, and whose responses to exposure may change with age.

*Environmental programming.* The process by which environmental conditions during sensitive developmental periods induce persistent changes in physiology, tissue organization, metabolism or gene regulation. Environmental programming is central to the DOHaD framework and can influence later disease susceptibility and functional reserve, although this does not necessarily imply a direct alteration of the intrinsic rate of ageing.

*Developmental stabilization*. The progressive transition from highly plastic developmental states towards more constrained biological configurations in which cell identities, tissue architecture and regulatory programmes become stabilized and regenerative potential is reduced. Stabilization does not imply the cessation of change, but a narrowing of the range of possible states. In the framework proposed here, this transition allows specialized and coordinated function while imposing constraints on plasticity, renewal and repair that may influence subsequent ageing trajectories.

*Reserve capacity*. The latent capacity of a cell, tissue or organ to maintain function despite stress, damage or loss. Reserve can derive from cell number, structural or functional redundancy, regenerative capacity, metabolic flexibility and compensatory mechanisms. It is partly established during development and subsequently modified by ageing and lifelong exposures.

*Resilience.* The capacity of a biological system to withstand a perturbation or to recover function after it. Resilience is related to, but distinct from, reserve capacity: reserve describes the resources or functional margin available to accommodate a challenge, whereas resilience describes the ability of the system to resist or recover from that challenge. Both depend on developmental configuration but remain dynamically influenced by maintenance, repair, adaptation and environmental exposure throughout life.

## 2. Development establishes the biological context of ageing

The most robust evidence linking development to later-life outcomes comes from early-life programming. Developmental conditions influence adult metabolism, cardiovascular function, endocrine regulation and vulnerability to disease. This literature is strongest where specific developmental exposures can be linked to durable physiological and epigenetic consequences. Prenatal undernutrition, placental insufficiency, maternal stress and endocrine disruption are among the best documented examples. The Dutch Hunger Winter remains one of the clearest human illustrations: prenatal famine exposure was associated decades later with altered metabolic and cardiovascular risk, and with persistent differences in DNA methylation at specific loci [8]. Similar principles emerge from animal models, where maternal protein restriction, glucocorticoid exposure or altered early nutrition can durably affect nephron number, pancreatic function, hypothalamic circuits and stress responses, thereby modifying later susceptibility to metabolic, cardiovascular and neuroendocrine disease [7,15].

Whether developmental perturbations also alter the intrinsic rate of ageing is more difficult to establish, as disease burden, reserve capacity—defined as the latent capacity of a tissue or organ to tolerate stress, compensate for loss or maintain function despite progressive damage—and biological ageing become tightly intertwined over time. Nonetheless, development establishes the physiological and structural conditions under which later decline occurs.

Development does so in several ways. It determines the number and distribution of cells within tissues, the size and composition of stem cell pools, and the architectural organization within which homoeostasis must operate [16]. It also sets endocrine thresholds and metabolic regimes that can remain remarkably stable across life. These features are not transient. They define the initial reserve, resilience and repair capacity of tissues, and therefore influence the conditions under which later damage accumulates or is tolerated. This reserve extends beyond cell number to include redundancy, metabolic flexibility and repair capacity, and it is therefore partly a developmental property.

## 3. Developmentally configured stem cell systems and ageing

Adult stem cells constitute one of the clearest biological bridges between development and ageing. Their pool size, lineage bias, quiescence depth, metabolic state and niche integration are largely established during development and maintained over time. The transition from fetal to adult stem cell states is not merely a change in tempo; it redefines their functional properties, as illustrated in haematopoiesis, where fetal haematopoietic stem cells are highly proliferative and expansion-oriented, whereas adult haematopoietic stem cells are more quiescent, more constrained and differently biased in lineage output [17,18].

Stem cell ageing is often discussed as a consequence of cumulative replication stress, DNA damage, inflammatory exposure or niche deterioration. However, it also reflects developmental configuration, as early established properties constrain altered responses. A highly quiescent stem cell system may protect against exhaustion and malignancy, yet respond less effectively to injury, whereas a more permissive system may regenerate better but incur other liabilities.

This is particularly evident in tissues with limited replacement. In the kidney, nephron endowment is largely fixed before or shortly after birth, and lower nephron number is associated with increased vulnerability to hypertension and renal decline later in life [19]. In skeletal muscle, the developmental establishment and subsequent transition to quiescence of satellite cells shape regenerative potential in adulthood [20,21]. In haematopoiesis, fetal and adult haematopoietic stem cells differ profoundly in proliferative behaviour, lineage bias and transcriptional regulation, and the fetal-to-adult transition has lasting consequences for how the blood system ages [17,18]. In the nervous system, early neurogenic tempo, neuronal subtype specification, circuit assembly and glial maturation set the long-lived architecture on which later ageing acts [22]. Even when development appears successful in a functional sense, it may leave tissues with different levels of reserve.

Stem cell niches reinforce this continuity. They are constructed during development, regulate stem cell behaviour throughout life and age in parallel with the stem cells they support [23]. Development therefore defines both the stem cell and the local system that governs its future function.

## 4. Epigenetic regulation across development and ageing

At the molecular level, development sets epigenetic landscapes that persist over long timescales. This is especially important for long-lived or post-mitotic cells, but it is not restricted to them. DNA methylation patterns, chromatin accessibility and transcription factor occupancy landscapes set during development contribute to maintaining lineage fidelity and cell identity long after development is complete [24–26]. Ageing-associated epigenetic drift [27–29] thus occurs not in a blank system but on top of a developmentally configured baseline. This point is central, because it establishes a mechanistic bridge between early developmental patterning and later-ageing trajectories.

Recent work gives this bridge greater molecular precision. A particularly interesting model proposes that during development and maturation, cell-identity transcription factors establish and maintain early-life regulatory regions, whereas later in life, stress-responsive factors, especially AP-1 family members, progressively gain access to alternative *cis*-regulatory elements [30]. This shift redirects transcription away from cell-identity programmes towards stress- and maturation-associated states. These findings suggest that ageing reflects the operation of shared regulatory systems under altered conditions of stress and chromatin accessibility, and that chronic or repeated stress may further accelerate this shift.

This view offers a mechanistic link between environmental stress, transcriptional drift and age-related cellular change. Consistent with this, oxidative- and nitrosative stress-dependent transcriptional alterations have been implicated in the progeroid disorder Cockayne syndrome [31]. In normal cells, the CSB protein exhibits altered promoter occupancy in response to oxidative stress [32], a process that is disrupted in CSB-deficient cells, as in Cockayne syndrome [33]. More broadly, other progeroid disorders display dysregulation of multiple reactive species, including reactive oxygen species (ROS), reactive nitrogen species and reactive sulfur species [34]. These species, conceptualized within the framework of the reactive species interactome, have also been implicated in ageing [35]. Together, these observations support a role for stress-dependent, epigenomically mediated transcriptional changes in progeroid phenotypes (see also next section) and raise the possibility that related mechanisms contribute to physiological ageing. Importantly, reactive species represent only one class of cellular stressors, and stress-responsive pathways, such as those involving AP-1, are activated by a broader range of stimuli. In this context, although the link between oxidative stress and ageing is well established [4], these findings emphasize the regulatory role of reactive species as modulators of transcriptional and chromatin states, rather than their canonical damaging effect.

## 5. Shared regulatory pathways in development and ageing

The reuse of molecular pathways across development and ageing reflects their evolutionary conservation. Pathways such as mTOR, insulin and insulin-like growth factor signalling (IIS), TGF-β, Wnt, Notch, Hippo, Hedgehog and FGF pathways all play central roles during development and later participate in tissue maintenance, regeneration, stress responses or age-related pathology [36]. Their deployment across life is not accidental. Most are evolutionarily ancient (table 1). mTOR and core insulin signalling components predate multicellularity, having originally functioned in unicellular contexts to regulate nutrient sensing, growth, metabolism and stress resistance [37,38]. Wnt, TGF-β and receptor tyrosine kinase-based signalling expanded or were assembled during early multicellularity in the metazoan ancestors to coordinate cell–cell communication, patterning and differentiation [39,40]. Notably, some components of these pathways have deeper evolutionary origins; for example, elements related to Wnt signalling may derive from more ancient metabolic systems, potentially linked to lipid metabolism [41], although their functional integration into canonical signalling pathways appears to have emerged in metazoans. Notch and Hedgehog are similarly tightly associated with metazoan tissue organization [42,43]. Although components of the Hedgehog pathway may have ancestral links to bacterial systems involved in lipid transport and homoeostasis [44], their role in cell–cell signalling pathways appears to be a metazoan innovation. By contrast, the Hippo pathway more clearly retains features of an ancestral unicellular system, combining ancient kinase modules with metazoan-specific developmental deployment [45]. Consistent with this, functional studies in close unicellular relatives of animals and early-branching animals suggest that the Hippo pathway originally functioned in cytoskeletal regulation and was subsequently co-opted to control proliferation and tissue size in multicellular contexts.

**Table 1.** Possible evolutionary origin and context-dependent roles of shared regulatory pathways in development and ageing.

pathway	possible origin	development	ageing
mTOR/IIS	ancestral unicellular systems: regulate nutrient sensing, growth, metabolism, stress resistance	embryonic growth and patterning	reduced mTOR extends lifespan (yeasts, worms, flies, mice)
IIS		essential for embryonic development (flies, mice)	reduced IIS extends lifespan (*Caenorhabditis elegans*, mice)
TGF-β	expanded in early multicellular metazoan ancestors: coordinates cell-to-cell communication, patterning, differentiation	development and homoeostasis	senescence, fibrosis, inflammatory remodelling, degenerative diseases
Wnt		embryonic patterning, stem-cell maintenance, polarity	either it preserves tissue integrity or promotes fibrosis/senescence
Notch	metazoan tissue organization	cell-fate decisions and tissue development	persistent signal is deleterious
Hippo	ancestral unicellular systems: ancient kinase modules; in metazoan: development	organ growth, proliferation and tissue architecture	its decline favours senescence and impaired tissue repair; its sustained activation: risk of uncontrolled growth and tumorigenesis
Hedgehog	metazoan tissue organization; ancestral links with bacterial lipid transport/homoeostasis?	patterning and cell-fate specification	stem cell maintenance/tissue regeneration; its decline contributes to reduced regenerative capacity

Specific examples illustrate how developmental functions and ageing functions intersect without being equivalent (table 1). mTOR is essential for embryonic growth and patterning, yet reduced mTOR signalling extends lifespan in yeast, worms, flies and mice, indicating that a pathway indispensable for development can become limiting for longevity once growth is complete [38,46–49]. IIS is lethal when strongly impaired during development in mice or flies, yet reduced IIS in adulthood can markedly extend lifespan in *Caenorhabditis elegans* and mice [50–53]. Wnt signalling regulates embryonic patterning, stem cell maintenance and polarity, but in ageing, it can either preserve tissue integrity or promote fibrosis and senescence, depending on context [54,55]. Notch is indispensable for fate decisions and stem cell regulation, but persistent or ectopic Notch activity in adult tissues can become deleterious [56,57]. TGF-β is crucial for development and homoeostasis, yet in ageing, it is increasingly associated with fibrosis, inflammatory remodelling and degenerative disease [58]. These pathways are not ‘pro-ageing’ or ‘pro-development’ in themselves. Their effects depend on the biological state in which they operate.

These observations are broadly consistent with established theories of ageing. For instance, hyperfunction models propose that the continued activity of developmental pathways beyond their optimal temporal window contributes to ageing-related pathologies [14]. Similarly, Dilman [59] proposed that mechanisms engaged during ontogenesis that ensure stability during development may later disturb homoeostasis once growth and development have ceased. An influential evolutionary framework relevant to this discussion is the theory of antagonistic pleiotropy. As originally formulated by Williams [60], antagonistic pleiotropy proposes that some genes or pathways exert beneficial effects early in life, while having deleterious effects later, reflecting the age-dependent weighting of their effects by the declining probability of reproduction. This concept remains important, as it captures the empirical observation that the same biological systems can have distinct consequences across life stages. However, rather than interpreting these patterns in terms of optimization for early-life fitness or as late-life effects being tolerated as a necessary cost, we emphasize that the same regulatory systems operate in biologically distinct states, transitioning from developmental plasticity to stabilized and constrained conditions in which stress and damage reshape their functional output.

Notably, analyses of cells derived from patients with progeroid Cockayne syndrome reveal a distinctive epigenomic signature that is not observed in related DNA repair disorders lacking progeroid features [61]. Strikingly, this signature is enriched in genes associated with developmental transcriptional programmes, including members of the *HOX*, *TBX*, *FOX*, *WNT*-associated, *GATA*, *SOX* and *PAX* families, which play central roles in embryonic patterning, cell fate specification and tissue organization. A subset of these regulators shows coordinated changes in DNA methylation and gene expression, suggesting that their deregulation is not merely correlative but functionally relevant. Importantly, several of these genes have also been implicated in physiological ageing, while others may represent less-explored components of developmental networks contributing to ageing phenotypes.

These observations support the idea that accelerated ageing, and possibly physiological ageing, can involve the inappropriate persistence, reactivation or misregulation of developmental programmes, consistent with models such as the hyperfunction theory [62], in which the continued activity of developmental and growth-related pathways beyond their optimal temporal window contributes to ageing-related phenotypes. Rather, these developmental pathways become progressively dysregulated through epigenetic drift, stress exposure or impaired maintenance mechanisms, potentially as a consequence of the constraints imposed by developmental stabilization itself.

Developmental pathways should therefore not be viewed as uniformly driving ageing. Rather, development establishes the biological context in which ageing occurs, while shared regulatory pathways may subsequently contribute to age-related changes, respond to accumulating stress and damage, or become dysregulated as maintenance declines. Their functional consequences are also shaped by the transition from developmental plasticity to more stabilized and constrained states. Thus, the involvement of developmental pathways in ageing does not imply a single direction of causality, but rather context-dependent interactions between regulatory activity, biological state and accumulating stress.

## 6. Comparative perspectives: development, lifespan and ageing trajectories

Comparative biology further supports the idea that development and ageing are linked through a broader organismal tempo. A first key observation is that mammalian lifespans vary by more than two orders of magnitude, from a few years in small insectivores to over two centuries in the bowhead whale, even after accounting for body size effects, as larger animals tend, on average, to live longer than smaller ones [63]. This diversity cannot be recapitulated by lifespan-extending interventions in short-lived model organisms [64], indicating that long lifespan arises from deeply embedded, species-specific biological configurations rather than from the modulation of a single pathway. Across mammals, lifespan correlates positively with developmental schedule, including gestation length and age at sexual maturity, in phylogenetically informed analyses [13,65,66]. Species that mature later often live longer, but this should not be interpreted as a direct causal effect of prolonged development on longevity. Rather, developmental duration, reproductive pace and somatic maintenance co-evolve as components of a coordinated life-history strategy.

At the same time, long-lived species do not necessarily generate less molecular damage. Across mammals, somatic mutation rates per year scale inversely with lifespan, suggesting that long-lived species accumulate genomic damage more slowly or manage it more effectively [67]. At the molecular level, comparative studies reveal recurrent associations between longevity and enhanced genome and epigenome maintenance, including improved DNA repair fidelity, chromatin stability and proteostasis, together with refined regulation of stress and inflammatory responses [64]. These adaptations vary across lineages, indicating that longevity reflects a distributed and context-dependent biological architecture. Together, these observations reinforce the view that ageing trajectories are embedded within broader organismal strategies that integrate developmental tempo, somatic maintenance, stress response and repair capacity. Development establishes the architecture, reserve and regulatory conditions within which ageing later unfolds.

## 7. Developmental stabilization, plasticity and ageing

Development proceeds from highly plastic states, characterized by proliferative capacity, flexible cell identity and extensive remodelling, to more constrained configurations in which cell fates are fixed and regeneration is reduced. This transition—developmental stabilization—represents a progressive narrowing of possible states. As plasticity declines and regenerative capacity becomes limited, organisms shift towards configurations optimized for coordinated function rather than remodelling, with important consequences for ageing. For example, developmental specialization establishes stable tissue architecture and cell identity but can restrict the plasticity and regenerative flexibility available to respond to later-life damage. This constraint reaches an extreme in highly specialized tissues such as the brain, where long-lived neurons and stable circuitry support specialized function but provide very limited capacity for cell replacement.

This perspective helps explain the association between sustained plasticity and attenuated ageing. In organisms such as cnidarians and planarians, regenerative capacity is maintained throughout life. The freshwater cnidarian *Hydra* remains a model of negligible ageing, although subsequent studies have shown that this phenotype is not universal and can vary with species, strain and environmental conditions [68–70]. This phenotype is associated with continuous self-renewal of multiple stem cell lineages and the absence of terminally differentiated, irreplaceable tissues [71,72]. Efficient autophagy is required to maintain epithelial stem-cell self-renewal and regenerative capacity in *Hydra*, and its experimental impairment is sufficient to induce ageing phenotypes [73,74]. In such a system, ongoing cell division and selection allow cellular damage to be diluted or eliminated. By contrast, in organisms composed of highly differentiated, poorly renewable cells within stable tissue architecture, comparable damage tends to accumulate over time and contribute to ageing (figure 1).

**Figure 1.**
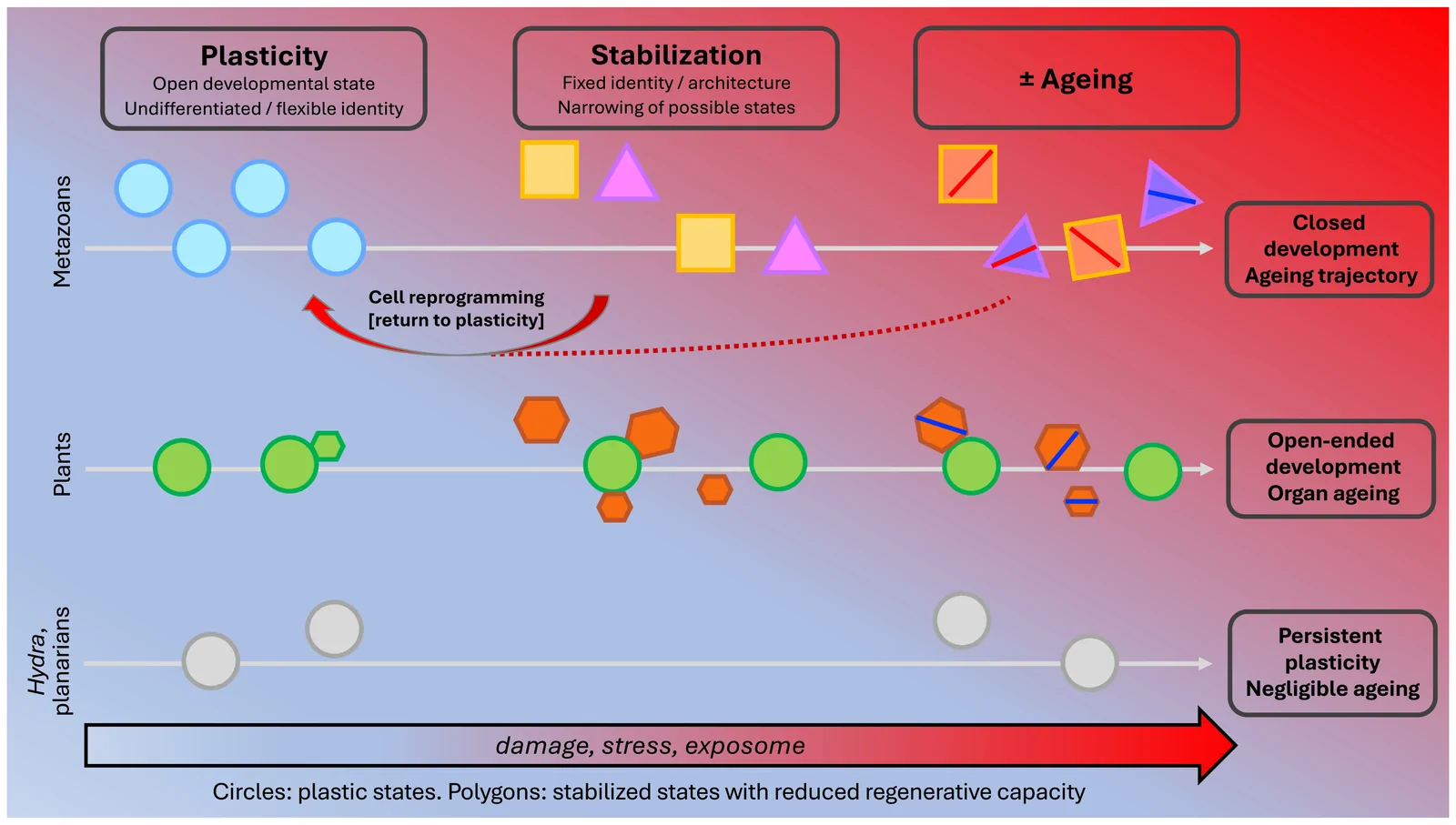
Developmental plasticity, stabilization and divergent ageing trajectories. Development progresses from plastic (circles) to stabilized states with reduced regenerative capacity (polygons). Ageing, resulting from stabilized states under cumulative stress, is indicated by damaged polygons (the line indicates damage). Plants maintain open-ended development and exhibit organ but not organismal ageing. *Hydra* and planarians, which retain regenerative plasticity, undergo negligible ageing.

Similarly, planarians maintain lifelong regeneration through persistent pluripotent stem cells [75]. The cnidarian *Turritopsis nutricula* can, under stress, revert from a sexually mature medusa-like adult to a juvenile polyp-like state and subsequently redevelop into the adult form [76]. Together, these systems maintain function through continuous renewal rather than long-term structural stability. Organisms such as *Hydra* may therefore attenuate classical ageing in part by retaining extensive plasticity and renewal rather than relying exclusively on long-term structural stability. Negligible ageing in these cases reflects the persistence of a development-like state.

By contrast, *Hydractinia symbiolongicarpus* provides direct evidence for a regenerative role of transient senescence: injury-induced senescent cells, through secretion of signalling factors resembling components of the senescence-associated secretory phenotype (SASP), promote the reprogramming of neighbouring somatic cells into stem cells required for whole-body regeneration [77]. Thus, these cnidarian examples do not imply a single ancestral mechanism linking regeneration and negligible ageing, but rather suggest that distinct mechanisms—including autophagy-dependent maintenance of stem-cell renewal and, in *Hydractinia*, senescence-induced cellular reprogramming—can preserve or restore the plasticity required for regeneration. These findings suggest that, at least in some cases, senescence-associated mechanisms may have a role in promoting plasticity and regeneration [78]. In more complex organisms, however, these mechanisms and their associated plasticity appear to be more constrained. The maintenance of stable cell identity and tissue architecture probably requires limiting plasticity, as unrestricted reprogramming would compromise structural integrity and increase the risk of malignant transformation. Consistent with this, related processes in mammals, such as senescence-associated or SASP-like signalling, can promote cell reprogramming, including in refractory tissues such as skeletal muscle [79], although in a more restricted and tightly regulated manner.

Cellular reprogramming in mammals illustrates that developmental plasticity is not irreversibly lost, but can be reactivated under appropriate conditions. Reprogramming somatic cells to pluripotency reverses multiple hallmarks of cellular ageing, including epigenetic age signatures, mitochondrial dysfunction and senescence-associated features [80,81]. Full reprogramming restores a state resembling early developmental plasticity [75], indicating that part of the ageing phenotype resides in reversible regulatory states. This does not negate damage accumulation, but shows that ageing is partly maintained by the regulatory landscape of differentiated cells.

The limitations of this process are equally informative. The loss of identity and tumorigenic risk associated with full reprogramming illustrate why such plasticity is normally constrained *in vivo* [82,83]. More broadly, pathways that promote growth, proliferation and regenerative plasticity during development can facilitate tumorigenesis when aberrantly activated later in life, whereas mechanisms that restrict these activities contribute to tumour suppression [84], but may also limit tissue renewal and repair during ageing. Consistent with this, partial reprogramming can ameliorate aspects of ageing without complete loss of cellular identity [85]. Together, these observations support the notion that developmental stabilization preserves function while contributing to the long-term conditions under which ageing emerges.

Developmental stabilization is not a universal explanation of ageing. Some long-lived organisms are highly specialized; some regenerative organisms still show decline; and many ageing phenotypes clearly depend on DNA damage, inflammation, mitochondrial dysfunction, proteostasis failure, endocrine drift and ecological pressures not reducible to development [4]. Nor is the relationship between stabilization and ageing binary. It is better understood as a continuum shaped by cell turnover, repair capacity, the loss of identity defences, niche dynamics, metabolism and organismal ecology. Even with these caveats, it remains difficult to ignore that organisms avoiding classical ageing do so not by escaping development, but by retaining varying degrees of regenerative plasticity and modular renewal. By contrast, organisms that achieve high functional specialization through developmental stabilization gain performance and robustness, but become more vulnerable to irreversible decline.

## 8. Plants and modular ageing

Plants provide a complementary perspective on the relationship between development and ageing. Unlike most animals, plant development remains open-ended throughout life, with continuous growth driven by meristematic stem cell populations.

Although plants undergo extensive development, many do not exhibit organism-wide ageing in the animal sense, as growth remains modular and meristem-driven throughout life. New organs are continuously produced from shoot and root apical meristems, while older modules may undergo senescence (here referred to as ageing) and be discarded. Leaves, for example, undergo a regulated ageing programme, while the organism as a whole can continue to grow and regenerate [86]. In perennials and many trees, this organization permits extreme longevity without a simple equivalent of the mammalian pattern of whole-organism decline [87–89]. Plant senescence is not merely deterioration; it is often a regulated developmental phase involving nutrient remobilization, hormonal control and programmed cell death. The key point is that development in plants remains open at the organismal scale even when individual organs stabilize and age. Ageing is therefore largely modular and organ-specific rather than organism-wide. This illustrates that extensive development is compatible with attenuated ageing when systems do not culminate in tightly stabilized, low-renewal states (figure 1).

This modular organization allows plants to achieve extreme longevity without the same degree of systemic ageing observed in animals. It reinforces the idea that ageing is closely linked to how development stabilizes biological systems. When development remains open and renewal is continuous, ageing is attenuated or spatially restricted. When development culminates in fixed architectures with limited renewal, ageing becomes more prominent at the organismal level.

## 9. Brain development and ageing

The brain provides a clear illustration of the relationship between development and ageing. It is constructed through tightly orchestrated processes of progenitor expansion, neuronal differentiation, migration, circuit formation and stabilization, after which many cells, especially neurons, are maintained for decades [90]. As a result, developmental decisions persist over exceptional timescales. This stability is essential for brain function, but it comes at the cost of limited regenerative capacity, as most neurons are long-lived and not readily replaced.

Development thus defines both brain function and long-term vulnerability. Development sets neural reserve, including neuronal number and identity, connectivity and metabolic capacity [91]. This reserve reflects the capacity of neural systems to sustain function despite stress, loss or pathology. Subtle developmental variations may therefore generate brains that function normally early in life yet are more vulnerable later. Emerging evidence suggests that some molecular systems involved in brain development may also contribute to neurodegeneration. For example, the amyloid precursor protein plays roles in neuronal development, including progenitor proliferation and synapse formation, and is also central to Alzheimer's disease pathology [92]. Although causal links remain to be fully established, these observations support the notion that components of developmental programmes may be redeployed or dysregulated in ageing contexts. Notably, this does not involve the classical, broadly acting developmental signalling pathways discussed above (§5), but rather more specific neurodevelopmental regulators, highlighting the emerging connection between neurogenesis and neurodegeneration.

Neuronal identity depends on stable chromatin landscapes and transcriptional programmes established during development and maintained over long periods. This stability is essential for function but also renders the brain sensitive to transcriptional drift. Recent work mentioned above, linking stress-responsive chromatin remodelling, AP-1 activity and erosion of cell-identity programmes [30], provides a mechanistic basis for how ageing may progressively destabilize these developmental regulatory states.

The brain is also highly sensitive to developmental environmental influences. Prenatal nutrition, stress, inflammation, endocrine changes and toxic exposures can alter neurodevelopment in ways that persist into adulthood, shaping later vulnerability through effects on reserve, metabolism and stress responses.

The brain therefore exemplifies how high specialization and stability allow function but limit repair. Damage, once accumulated, is difficult to reverse. In this context, brain ageing reflects the long-term consequences of maintaining highly stabilized, weakly renewable systems under cumulative stress and damage.

## 10. Limits and scope of the developmental framework

### (a) Are all ageing-related mechanisms necessarily rooted in developmental history?

Not all mechanisms contributing to ageing necessarily originate in developmental processes; rather, we propose that developmental history can determine how strongly their consequences are expressed and whether they can be compensated.

Are all ageing-related mechanisms necessarily rooted in developmental history? Let us consider, for instance, the hierarchy proposed by López-Otín *et al.* [4], which distinguishes primary, antagonistic and integrative hallmarks of ageing. Our perspective suggests a different interpretation of this hierarchy. Consider DNA damage, classified as a primary hallmark: DNA lesions occur throughout life and also affect stem and highly regenerative cells, but their consequences depend critically on what the cell or tissue can do with them. A plastic or renewing system can repair damage, eliminate damaged cells and replace them, or dilute damage through cell division, whereas highly differentiated, long-lived cells have progressively fewer such options. Thus, DNA damage may become a major driver of ageing not simply because it accumulates, but because developmental commitment progressively restricts the capacity to cope with its consequences. The same principle can be extended to the other primary hallmarks: telomere attrition ultimately limits proliferative and regenerative capacity; epigenetic alterations occur within, and can sometimes be reset by reprogramming of, the differentiated state; and loss of proteostasis or macroautophagy becomes particularly consequential in long-lived cells that cannot readily dilute damage or be replaced.

The antagonistic hallmarks are even more closely intertwined with developmental programmes: nutrient sensing and mitochondrial metabolism regulate growth and cell-fate transitions, while senescence itself operates during normal embryonic morphogenesis, where senescent cells are cleared and tissues remodel [93,94].

Finally, among the integrative hallmarks, stem-cell exhaustion should perhaps not be regarded simply as a downstream consequence of ageing, but as a causal gate that determines whether upstream damage can still be compensated; altered intercellular communication similarly compromises the developmental organization and regenerative niches required to maintain tissues. Chronic inflammation and dysbiosis are less directly rooted in differentiation, but their persistence and pathological consequences are strongly influenced by declining tissue repair and regenerative resolution.

Thus, rather than constituting a linear cascade from primary damage to integrative consequences, the hallmarks may acquire their ageing-promoting significance within a developmental trajectory in which increasing specialization is accompanied by a progressive restriction of cellular and tissue plasticity.

Cnidarians further illustrate how different mechanisms can sustain a highly plastic, regenerative state associated with negligible or limited ageing, including autophagy-dependent maintenance of stem-cell renewal in *Hydra* [71–74] and senescence-induced cellular reprogramming in *Hydractinia* [75].

### (b) Quantitative dimension of developmental constraints and ageing

The relationship is also not binary. Whether stress produces adaptation, dysfunction or irreversible decline depends on its magnitude/duration relative to the compensatory capacity of the developmentally configured system.

Importantly, this relationship is not absolute but quantitative. Developmental history may define the repertoire and thresholds of responses available to a cell or tissue, while the magnitude and duration of stress determine which response is engaged. ROS provide a particularly clear example, as different levels of redox signalling can support self-renewal, proliferation or differentiation, whereas stronger or sustained oxidative stress can impair stem-cell function and promote senescence or cell death [34,95]. This principle extends well beyond ROS: increasing DNA damage can shift cellular responses from repair to persistent arrest, senescence or death [96]; proteotoxic/ER stress activates adaptive responses until the capacity to restore proteostasis is exceeded [97]; and moderate mitochondrial stress can induce protective responses, whereas more severe or persistent dysfunction becomes detrimental [98]. Similarly, telomere shortening becomes critical only beyond thresholds that activate persistent DNA-damage responses, while transient senescence or inflammation can contribute to tissue remodelling and regeneration but become deleterious when persistent or insufficiently resolved [99].

Thus, the critical variable may not be the absolute level of stress or damage, but its magnitude relative to the system's capacity to compensate for it. The same molecular burden may remain functionally neutral in a plastic, regenerative context but become ageing-promoting when developmental commitment and declining regenerative capacity reduce the available compensatory reserve.

Ageing may therefore involve the progressive crossing of stress thresholds whose position is itself conditioned by developmental state and remaining adaptive and regenerative capacity; rather than simply erasing developmental constraints, increasing stress may overwhelm the capacity of a given developmental state to maintain or restore homoeostasis and, in some contexts, drive transitions towards alternative cellular states.

## 11. Conclusions

Development establishes durable biological constraints that shape ageing trajectories. Ageing unfolds within structures, cell populations and regulatory networks configured during early life. The relationship between development and ageing is therefore not simply sequential, but deeply interconnected. Development defines the conditions under which ageing occurs, including the balance between plasticity and stability, regeneration and preservation, flexibility and constraint.

Ageing is not a continuation of development, but the consequence of maintaining developmentally stabilized systems under stress and damage. When systems retain plasticity and regenerative capacity, ageing is attenuated; when they are highly specialized and weakly renewable, ageing becomes more prominent.

This perspective does not negate the role of molecular damage, stress responses or environmental influences, but situates them within a broader developmental framework. Damage accumulates, but its consequences depend on the properties of the system in which it occurs, properties largely defined by development (figure 2).

**Figure 2.**
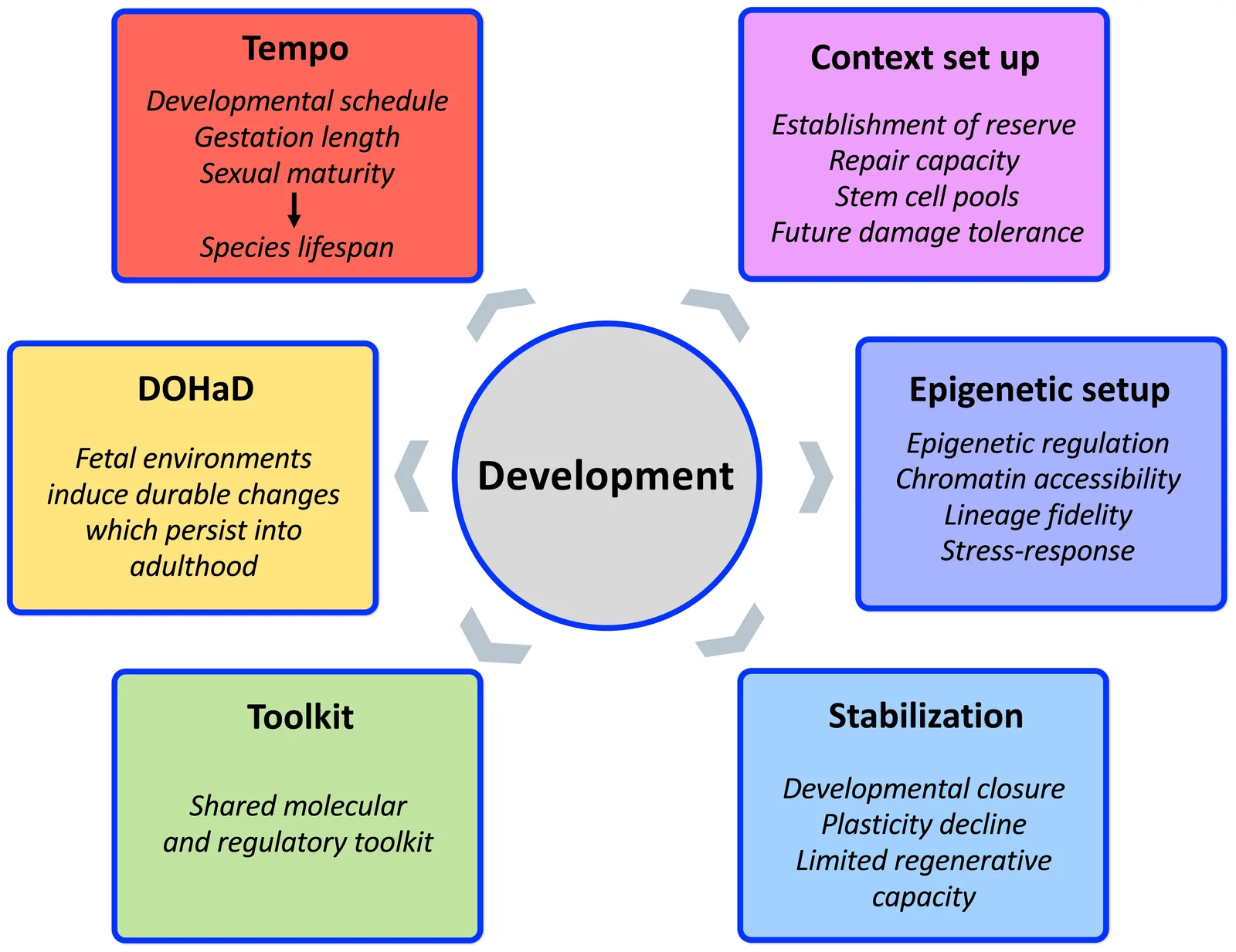
Multi-level influence of development on ageing. Development influences ageing across multiple layers, from early-life effects persisting into adulthood, in the framwork of DOHaD, to the establishment of developmental tempo and thereby longevity trajectory; the determination of tissue reserve, stem-cell pools and repair capacity; the epigenetic landscape; the progressive stabilization of biological systems (closure of development) associated with reduced plasticity; and shared molecular and regulatory toolkits.

The brain provides a particularly clear example of this relationship. Its development establishes both functional capabilities and the constraints that shape its long-term behaviour. High specialization and stability allow complex function, but limit renewal and increase vulnerability to cumulative stress.

More broadly, ageing can be understood as the long-term outcome of maintaining systems that have been optimized for function at the expense of flexibility. In this sense, ageing reflects both the accumulation of damage and the progressive limitation of biological systems constrained by their developmental history.

## Data Availability

This article has no additional data.

## Theme

One contribution of 21 to a theme issue ‘Developmental circuits in ageing’.
